# Holding-on: co-evolution between infant carrying and grasping behaviour in strepsirrhines

**DOI:** 10.1038/srep37729

**Published:** 2016-11-24

**Authors:** Louise Peckre, Anne-Claire Fabre, Christine E. Wall, David Brewer, Erin Ehmke, David Haring, Erin Shaw, Kay Welser, Emmanuelle Pouydebat

**Affiliations:** 1 UMR 7179 C.N.R.S/M.N.H.N., 57 rue Cuvier, Case postale 55, 75231, Paris Cedex 5, France; 2 Department of Evolutionary Anthropology, Duke University, Durham, North Carolina, 27708, USA; 3 Duke Lemur Center, Durham, North Carolina, 27705, USA

## Abstract

The origin and evolution of manual grasping remain poorly understood. The ability to cling requires important grasping abilities and is essential to survive in species where the young are carried in the fur. A previous study has suggested that this behaviour could be a pre-adaptation for the evolution of fine manipulative skills. In this study we tested the co-evolution between infant carrying in the fur and manual grasping abilities in the context of food manipulation. As strepsirrhines vary in the way infants are carried (mouth vs. fur), they are an excellent model to test this hypothesis. Data on food manipulation behaviour were collected for 21 species of strepsirrhines. Our results show that fur-carrying species exhibited significantly more frequent manual grasping of food items. This study clearly illustrates the potential novel insights that a behaviour (infant carrying) that has previously been largely ignored in the discussion of the evolution of primate manipulation can bring.

Prehension, referring to movements in which an object is seized and held partly or wholly by an organ, is widespread among tetrapods^1^. The three main modes of grasping among tetrapods are oral, manual, and pedal prehension. Mammals appear to be, in general, more dexterous in manual grasping and handling than other vertebrates^1^. In primates, hand use is a key behaviour during locomotion, foraging, the manipulation of objects, and in social interactions^2^. However, manual grasping is generally thought to be associated primarily with feeding behaviour^1^,^3^,^4^. Although humans have long been considered as possessing the greatest dexterity during manual grasping^2^, all primates show the ability to grasp food and many of them use a variety of grip types^5^,^6^,^7^. Moreover, even among primates, food manipulation is associated with different ecological contexts and morphologies; hence, the uniqueness of the human hand only exists along a morphological and behavioural continuum^2^. Current patterns of manual grasping abilities and hand use are the result of evolutionary processes induced by potentially multiple selective pressures operating in different ecological and behavioral contexts.

The “arboreal hypothesis” suggests that prehensile and sensitive hands evolved first in association with the adoption of a more arboreal lifestyle^8^,^9^,^10^. However, other hypotheses suggest that arboreality itself does not necessary leads to grasping abilities and focus on the combined roles of arboreality and predation. Hence, the “visual predation hypothesis”^11^ proposes a visually guided manual predation of insects on fine branches as a driver while the “angiosperm exploitation hypothesis”^10^ suggests that the exploitation of fruit and flowers on the terminal branches was the most important ecological innovation that initially led to the evolution of the distinctive primate morphology^9^. These three main hypotheses on the origin of grasping abilities overlap in giving great importance to the selective pressures associated with the arboreal environment.

However, although selection for fine branch foraging, food properties, and predation may be sufficient to explain the origin of primate grasping, it does not explain by itself the further evolution of enhanced hand dexterity and its variations among primate species^2^,^12^. Hence, it is of importance to distinguish the factors associated with these variations in hand dexterity or hand use to identify the possible relevant selective pressures that more specifically led to such dexterous and accurate forms of grasping.

Bishop^13^, also known under her married name Jolly^14^,^15^, emphasized the importance of fur-grasping for the young to hold onto their mother. She further described this behaviour as involving a close contact between the distal phalanges of the digits, and argued “that some such focus of control on the touch-pads is a likely forerunner of fine control of the hand”^13^. Hence, she suggested that this grip could potentially be the evolutionary precursor to the development of fine manipulative skills with true fine control of the hand involving accuracy.

Infant carrying is one of the most obvious features of maternal behaviour in most mammals and is crucial for the survival of the young^16^. In the majority of primates the young are not left unattended during foraging but instead are carried by their mother^17^,^18^. Surprisingly, this behaviour has received relatively little attention^19^,^20^. Some variation between species exists, however, and two main patterns of infant carrying can be distinguished^16^. In some species, the young are relatively altricial, appear incapable of coordinated movement, have their eyes closed, and are typically maintained in a nest or a tree-hole for several weeks^16^,^21^. This behaviour is usually associated with occasional oral carrying. This pattern has only been reported for *Simias concolor, Presbytis potenziani, Cebuella pygmaea* and *Procolobus verus* among anthropoids, but is also found in several strepsirrhines^16^,^22^. In other species, infants are born with grasping extremities enabling them to cling to the mother’s fur efficiently from birth onward^13^,^19^,^20^,^21^. This pattern typifies most apes and monkeys and some strepsirrhines, including all of the *Eulemur* species^16^,^18^ and Lorisidae.

As oral transport is widespread among nest living species such as rodents, carnivores and insectivores, this behaviour is probably primitive^16^,^18^,^23^. Using parsimony-based methods to reconstruct the phylogenetic history of traits, Kappeler (1998) showed that the ancestral primate was likely nocturnal and solitary, producing a single young that was first kept in a nest and subsequently carried by the mother in the mouth to be ‘parked’ outside the nest^21^. In species with strong environmental constraints such as arboreal or flying species, selection against nesting (to avoid nest predation and nest parasites) presumably led to the evolution of infant carrying because the infant is not able to follow his mother during foraging^16^.

In primates fur-clinging is supposed to have evolved several times independently^19^; once in the common ancestor of anthropoids and four times in strepsirrhines (in the Lorisidae, the Lemurinae, the Indridae and possibly in *Phaner furcifer*^16^), which suggests that the costs are balanced and that it probably presents significant advantages^17^. Unlike oral carrying mothers, when the young is clinging to the fur, the mother’s body has no significant active role in infant carrying apart from supporting the extra load of the infant on its limbs. The mother can thus benefit from the liberation of her mouth and hand(s) and can pursue other activities such as foraging while ensuring constant protection and thermoregulation to her young. Hence, young holding on to their mother’s fur will also have social, physiological, and protective benefits.

In the present study we tested for a possible evolutionary link between infant carrying behaviour and hand dexterity. As strepsirrhines show both oral infant carrying and fur grasping (Fig. 1), they represent an excellent model to test this hypothesis. In addition, their position near the base of the primate tree may contribute to a better understanding of the factors driving the evolution of dexterous grasping ability in humans and other anthropoid primates.

## Results

**Figure 2:**
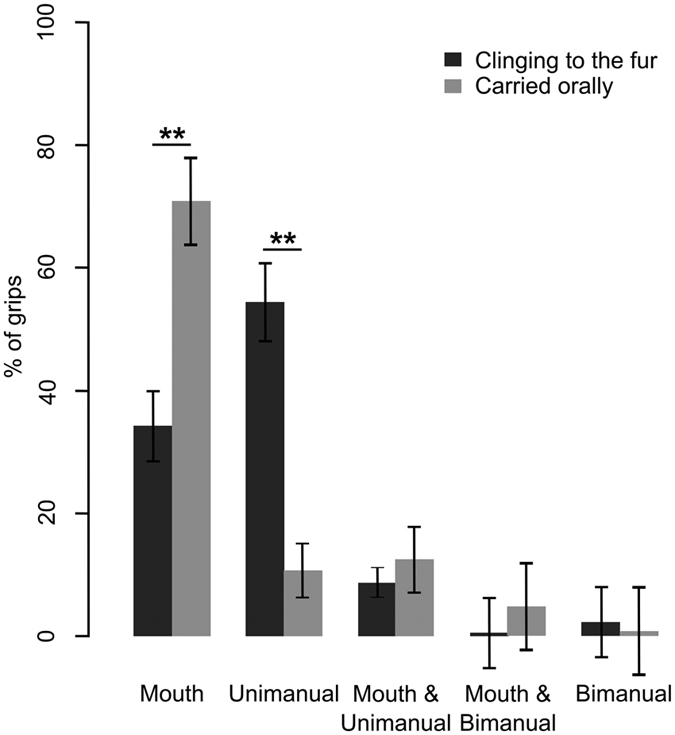
Mean proportion of the different grip types used to grasp big and hard items in relation to infant carrying (fur: N = 15, N_ind_ = 53, N_grip_ = 1432; mouth: N = 6, N_ind_ = 24, N_grip_ = 497; Mann-Whitney U tests; **P < 0.01). Data are represented as mean ± SEM.

As it is know that food size and mobility impact grasping^24^ we here analysed only grasping of large and hard static food items. A MANOVA performed on the transformed proportion of the different grip types while grasping big and hard items (Table S1) indicated a significant effect of infant carrying (Wilks’ λ = 0.47, F_1, 15_ = 3.43, *P* = 0.029). In order to grasp big and hard food items, fur-clinging species were observed to use significantly less mouth grips during feeding (36 ± 6%, *N* = 16, *N*_ind_ = 53, *N*_grip_ = 1432) than oral-carrying species (71 ± 7%, *N* = 6, *N*_ind_ = 24, *N*_grip_ = 497) (Mann-Whitney U test: W = 10, *P* = 0.005; Fig. 2). Moreover, fur-clinging species were observed to use significantly more unimanual grips during feeding (52 ± 7%, *N* = 15, *N*_ind_ = 53, *N*_grip_ = 1432) than oral-carrying species (11 ± 5%, *N* = 6, *N*_ind_ = 24, *N*_grip_ = 497) (Mann-Whitney U test: W = 83.5, *P* = 0.003; Fig. 2). No significant differences were observed between the two groups regarding the proportion of combined oral and unimanual grips (Mann-Whitney U test: W = 41, *P* = 0.78; Fig. 2), oral and bimanual grips (Mann-Whitney U test: W = 29.5, *P* = 0.143; Fig. 2), or bimanual grips (Mann-Whitney U test: W = 59.5, *P* = 0.23; Fig. 2). Bimanual grips are rarely used by lemurs (Fig. 2) and will not be discussed further.

The proportion of unimanual, combined oral and unimanual and combined oral and bimanual grips exhibited a significant phylogenetic signal (Table 1). This suggests that closely-related species have a more similar behavioural pattern. A phylogenetic MANOVA was performed on the transformed proportions of the different grip types used to grasp big and hard items to test whether infant-carrying behaviour impacts food manipulation when taking into account phylogenetic relationships. When considering phylogeny, the effect of infant carrying behaviour (*P* = 0.53) is no longer significant suggesting that the use of grip types and infant carrying behaviour tend to be similar in species that are closely related.

## Discussion

Although selection for fine branch foraging, food properties, and predation may be sufficient to explain the origin of primate grasping^8^,^9^,^10^,^11^, it does not explain by itself the further evolution of enhanced hand dexterity and its variations among primates^2^,^12^. Hence, it is of importance to distinguish the factors associated with these variations in hand dexterity or hand use to identify the possible relevant selective pressures. This study investigated the relationship between infant carrying strategy (in the mouth or clinging to the fur) and manual dexterity (measured by the relative proportion of hand-grip type used to grasp immobile food items) across 21 strepsirrhine species. These data were collected in an effort to test for a possible evolutionary link between these two behaviours. Our results showed, firstly, important differences in hand use propensity between strepsirrhines during a non-constrained task reflecting differences in hand dexterity. Moreover, we found a link between the propensity to use the hand to grasp food items and the infant carriage pattern of the species. Fur-clinging species used significantly more unimanual grips (5 times more) and significantly less mouth-grips (two times less) than oral-carrying species. This result confirms a potential evolutionary link between fur-grasping and hand dexterity in primates.

**Figure 3:**
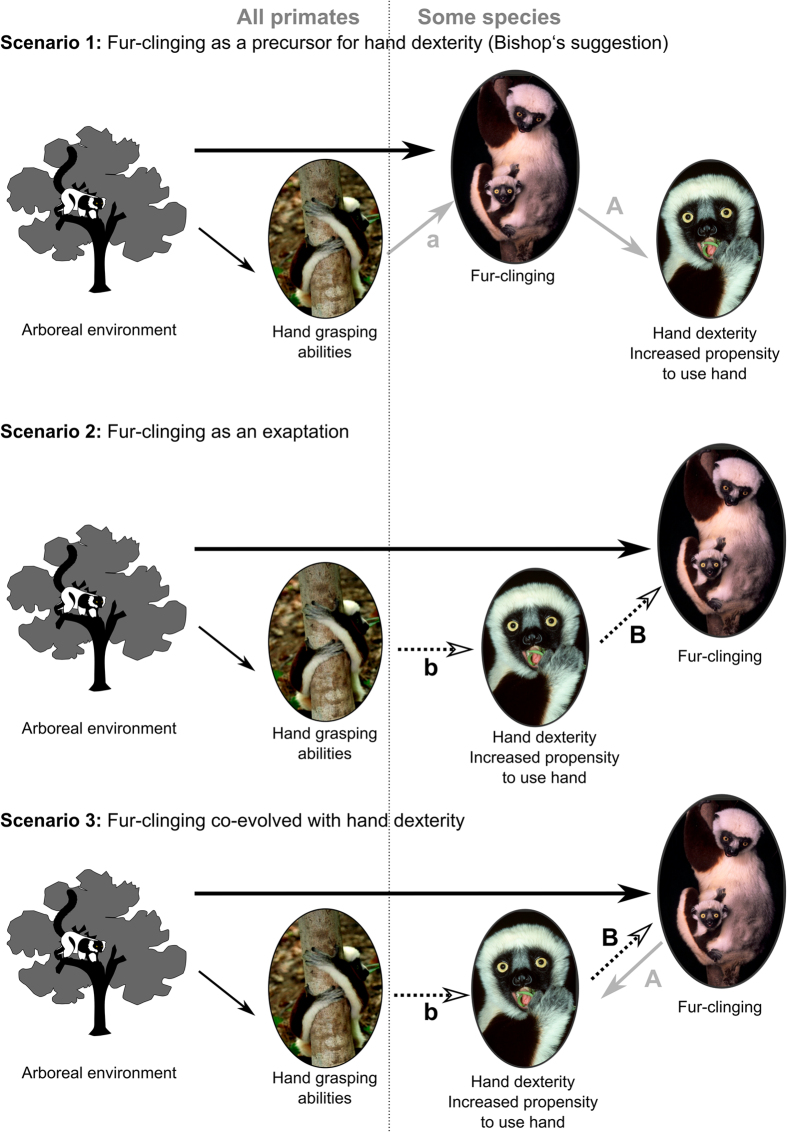
Schematic representation illustrating the three possible evolutionary scenariosas described in the discussion. Pictures of Coquerel’s sifaka (*Propithecus coquereli*); photo credit David Haring.

**Figure 4:**
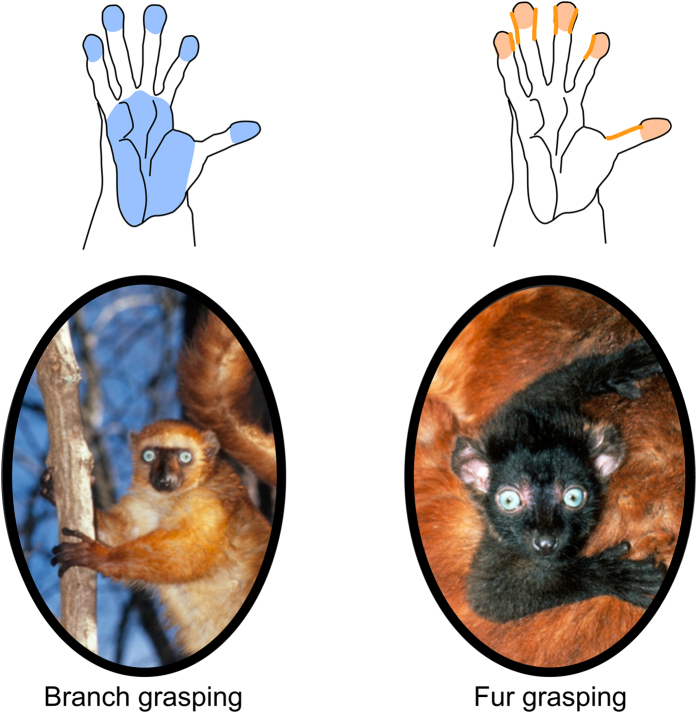
Different surface areas of the hand involved in the grips used to grasp branches during locomotion (blue surface in the representation of a hand), and when the infant grasps its mother fur (blue surface in the representation of a hand). Pictures of blue-eyed black lemur (*Eulemur flavifrons*); photo credit David Haring.

**Figure 5:**
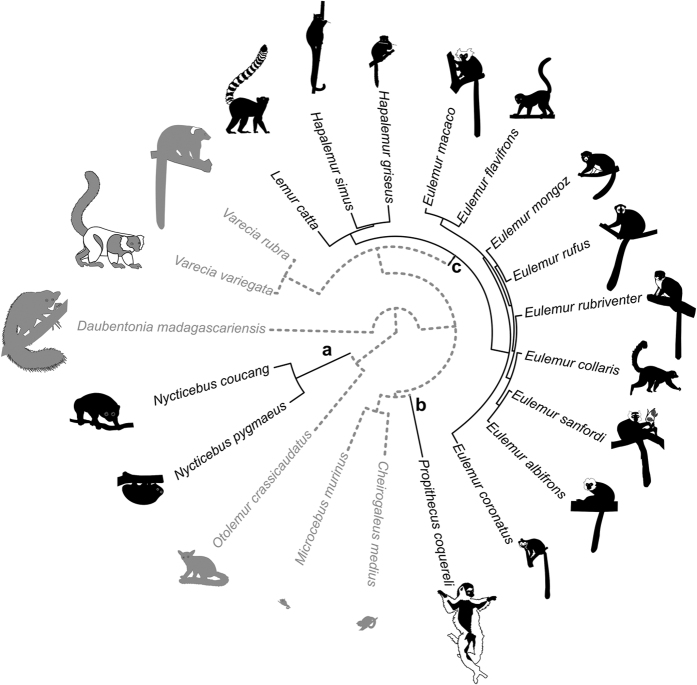
Consensus tree of the studied species (from the 10kTrees Project version 3) 43. Light grey color represents species that carry their infant orally whereas black color represents species for which infants grasp the fur of their mother. Three independent origins of ‘fur-grasping’ are present: (**a)** the independent origin for the loris clade (*Nycticebus coucang* and *N. pygmaeus*); (**b)** the independent origin for the Coquerel’s sifaka (*Propithecus coquereli*) and, (**c**), the independent origin for the brown lemurs (*Eulemur sp.*), bamboo lemurs (*Hapalemur sp.*), and ringtail (*Lemur catta*).

Based on these results three evolutionary scenarios can be imagined (Fig. 3). The first scenario (Fig. 3 Scenario 1) fits to Bishop’s suggestion that fur-grasping was a potential precursor for enhanced manipulative skills in primates^13^. The grip used by young lemurs to hold on the mother is a grip type with each finger pressed toward the next and the fingertips pressed toward the palm^13^. Hence, for all species analysed, fur-gripping depends on the close contact between the distal phalanges of digits two to five and the second phalanx of the first digit, hence, engaging different contacts than those imposed by the gripping of branches involving the whole palm and all palmar parts of the fingers^25^ (Fig. 4). Bishop considered this particular fur grip as a “direct approximation of touch-pads” constituting therefore an interesting potential “forerunner of fine control of the hand”^13^. Hence, specific selective pressures (e.g. nest parasites and nest predator avoidance) could have led some species to continually cling to their mother’s fur, developing in this way the use of the hand for fine grasping (Fig. 3 Scenario 1A). These dexterous abilities could then have been more frequently expressed later on in other contexts such as foraging. This hypothesis is supported by the fact that once it has evolved, fur grasping was conserved in nearly all lineages, possibly because of behavioural and physiological co-adaptations^16^. Moreover, even if in most of the human societies infants do not grasp their mother but are rather held actively by her or a related person^26^,^27^, touching the palm of a baby’s hand readily elicits a reflex called a “palmar grasp reflex” or “clinging reflex”. This reflex consist of the flexion of all the fingers around the elicitor’s finger followed by a clinging phase^28^. This reflex allows newborn primates to support their own weight for several minutes when holding onto a horizontal rod^29^,^30^. This reflex is likely phylogenetically primitive, supporting even more strongly the fact that fur-grasping could be a potential precursor for enhanced manipulative skills in humans and primates in general. However, previous studies have shown that fur-carrying has likely evolved several times independently from a mouth carrying ancestor^16^,^18^ (Fig. 5). This suggests that in these species the young were pre-adapted to cling. A more prehensile and sensitive hand may, indeed, constitute a prerequisite condition for the young to be able to cling to the fur of their mother (Fig. 3 Scenario 1a). Strepsirrhine species are all predominantly arboreal and, therefore, submitted to similar selective pressures, which are in all likelihood responsible for rendering the hand more sensitive and prehensile. Nevertheless, the gripping of branches is quite different than the gripping of fur and a more specific pre-adaptation could be needed.

In this way, another scenario (Fig. 3 Scenario 2) suggests that fur-grasping is an exaptation with species having developed increased manual dexterity, using it to reduce the constraints associated with nesting and benefiting from a close carrier-young association (Fig. 3 Scenario 2 B). In this scenario, other selective pressures associated for instance with the diet or the habitat should be responsible for selecting an increased manual dexterity (Fig. 3 Scenario 2b). This scenario is, however, difficult to defend regarding the tight link that exists between these two behaviours. Indeed, we would expect to find some species having developed increased hand dexterity without necessary using it for infant carriage. Instead, we observe in our results a considerable difference in hand usage between the two groups and relatively small differences between species within the orally-carried species group (SEM = 5%).

A third scenario (Fig. 3 Scenario 3) would offer, combining elements from the two previous ones, a more complex but more plausible scenario. This last scenario considers a co-evolution between hand-dexterity and fur-clinging. Some ecological parameters could impose selection on the hand-grasping abilities of the different species, increasing at least slightly hand dexterity in all species (Fig. 3 Scenario 3b). This idea is supported by the fact that all species studied were observed to grasp food items with their hand(s). This slightly enhanced hand dexterity providing a sufficient pre-adaptation to be later used by the young of some species to grasp their mother’s fur (Fig. 3 Scenario 3B). Finally, fur-clinging could, in turn, have reinforced the ease and habit to use the hand leading to the observed differences in hand dexterity between the species (Fig. 3 Scenario 3A). Hence, this co-evolution scenario offers a more plausible explanation for the pre-adaptation and fits with our observations: all species used their hands to some degree for grasping, yet, important difference exist between animals with different carrying strategies.

Interestingly, while dexterous hand use has long been recognized among a suite of other anatomical and behavioural traits that distinguish primates from other mammals^11^,^31^,^32^,^33^, long-term infant carrying while foraging is similarly relatively rare in eutherian mammals, except among primates^16^,^23^. Hence, this tight link seems to exist even in a broader scale. In this way, catarrhine species, known to show the highest degree of variety and accuracy in grasping behaviours, are all fur clingers (with the exception of the thumbless, oral carrier, *Procolobus verus*). Among platyrrhine species, more variations between species seems to exist in hand use propensity and more studies have to be conducted to discuss the relation between these variations and the one existing in the infant carrying strategies particularly dorsal versus ventral infant carrying^19^.

To conclude, our study illustrates the potential novel insights brought by a behaviour (infant carrying) that has previously been largely ignored in the discussion of the evolution of primate hand dexterity. The grasping ability of hands and feet which were acquired in a common ancestor in response to moving and foraging in trees could have been followed in some species by a co-evolution between hand grasping dexterity and fur-clinging. To further investigate this hypothesis it would be interesting to explore the differences in infant carrying patterns and to better describe and compare the mechanical and anatomical aspects of the grip in different fur-clinging species across primates as a whole.

## Methods

### Sample

We collected data for 77 individuals of 21 different species of strepsirrhines comprising six of the seven strepsirrhine families (Cheirogaleidae, Daubentoniidae, Indriidae, Lemuridae, Galagidae and Lorisidae), excluding Lepilemuridae. Nearly all data were collected at the Duke Lemur Center (Durham, North Carolina, United States) where housing conditions and enrichment are similar for all species. The only species that was not housed in similar conditions was *Hapalemur simus* which is housed at the Vincennes Zoo (Paris, France). These individuals constitute a sample of 38 females and 39 males with ages ranging from 1.6 to 35.7 years old (mean age 16.0 ± 1.2 years old). Animal handling was performed in compliance with the International Primatological Society (IPS) Guidelines for the Use of Nonhuman Primates in Research according to the protocol #A089–14–04 approved by the Duke University Institutional Animal Care and Use Committee.

### Behavioural data collection

We videotaped each individual in its home enclosure during five days during their normal feeding period. We used digital video cameras (SONY HDR-PJ790V, full HD 1080, 24.1MP; SONY HDR-SR11, 10.2MP; SONY Handycam, HDR-PJ230, 8.9MP; SONY HDR-CX240E, full HD 1080, 9.2MP) for the diurnal species and a low light digital video camera (SONY HDR-SR11 10.2MP) for the nocturnal species. The usual diet constituted of different food items including raw pre-cut pieces of fruits and vegetables as well as monkey chow (Labdiet Monkey Diet Jumbo Constant Nutrition^®^ and ZuPreem^®^ Primate Dry Diet). Although insects are part of the diet for some species we did not analyse manual prehension for these items. As a previous study showed that the properties (size and consistency) as well as mobility of the food item influenced the grasping strategy adopted^24^, we decided to control for food size and hardness to account for any potential bias in manipulative activity across species (Table S1). As small and soft items were grasped mainly with the mouth by all species, they do not constitute a good model to analyse manual prehension. We considered an item as big when bigger than one hand width of the focal species and as hard when it imposed a significant resistance to the teeth (defined as at least as hard as cucumber in our range of items). Here, we thus analyzed only the grasping of large static items.

We analysed the videos using Avidemux 2.6.8 (Free Software Foundation, Inc.). We annotated every instance of identifiable big and hard item grasping, leading to a total of 1929 grips for 102.06 hours of video analysed. The mean number of grips recorded per individual was 25 ± 2 grips (range from 5 to 86). Grip types were characterised by the body part(s) involved as oral, unimanual, bimanual, combined oral-unimanual, or combined oral-bimanual. We included in the dataset only individuals observed for at least 5 grip events.

### Infant Carrying behaviour

We used the literature to characterize the infant carrying behaviour of each species (Table 2). Some species were observed to perform both types of behaviour, however, one of these patterns is always dominant. Hence, we classified species in two exclusive categories, “oral carriers” or “fur clingers” based on the most commonly observed strategy^33^,^34^,^35^,^36^,^37^,^38^.

### Statistical Analyses

We analysed the data using R3.0.2 (R Core Team^39^). We arcsine transformed the proportions of the different grips used. Descriptive statistics (means ± standard error of the mean) were calculated for each individual and each species. In this study, “*N*” was used to denote the number of species whereas “*N*_ind_” was used to denote the number of individuals and “*N*_grip_” the number of grasping events.

To assess the effect of infant carrying on the proportions of grip types, we first analysed the behavioural dataset using a MANOVA (Multivariate Analysis of variance). We then performed post-hoc Mann-Whitney U tests in order to determine the types of grip influenced by infant carrying.

Strepsirrhines share their phylogenetic history and, therefore, they cannot be considered as independent data points^40^. To address this issue and determine the adequacy of using conventional or phylogenetically informed statistical analyses we tested the presence of a phylogenetic signal^41^ using the R “phylosig” function included in the “phytools” package (version 0.4–45)^42^. Pagel’s λ is a scaling parameter for the correlations between species traits, relative to the correlation expected under Brownian motion. Values of λ < 1.0 correspond to traits being less similar among species than expected based on their phylogenetic relationships. This method requires the use of a phylogenetic tree. We used a consensus tree in version 3 of the 10kTrees Project^43^ with the species for which we had behavioural data.

We performed a phylogenetic Multiple Analysis of Variance (MANOVA) using the R “aov.phylo” function included in the “geiger” package (version 2.0.3)^44^. We performed one thousand simulations of character evolution taking the phylogenetic relationships into account and we run a MANOVA on these simulated trait values to create an empirical null distribution of F-statistics. Finally, we compared the 95^th^ percentile of the null distribution with the results of the traditional MANOVA. Values are considered statistically significant if the non-phylogenetic F_trad_-value was greater than the critical F_phyl_-value of the empirical F-distribution.

## Additional Information

**How to cite this article**: Peckre, L. *et al*. Holding-on: co-evolution between infant carrying and grasping behaviour in strepsirrhines. *Sci. Rep.* **6**, 37729; doi: 10.1038/srep37729 (2016).

**Publisher’s note:** Springer Nature remains neutral with regard to jurisdictional claims in published maps and institutional affiliations.

## Supplementary Material

Supplementary Information

## Figures and Tables

**Figure 1:**
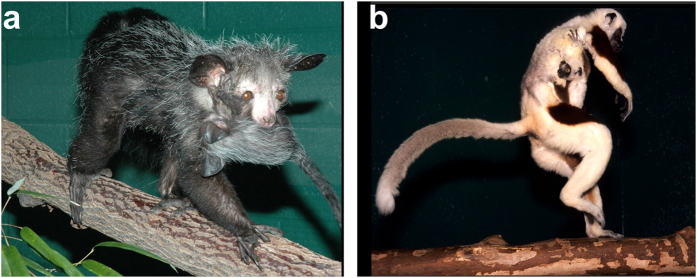
Pictures showing: (**a**) oral infant carrying behaviour as illustrated by the aye-aye (*Daubentonia madagascariensis*); (**b**) fur grasping by infant as illustrated by Coquerel’s sifaka (*Propithecus coquereli*). Photo credit David Haring.

**Table 1:** Phylogenetic signals in grasp types.

Grip type	Pagel’s λ	*p*
Mouth	0.7	0.053
Unimanual	0.76	0.002**
Mouth & Unimanual	0.58	0.03*
Mouth & Bimanual	0.98	0.016*
Bimanual	0.00006	1

^*^P < 0.05; **P < 0.01.

**Table 2:** Description of the infant carrying pattern and common litter size of the studied species and their sample size in the study.

Species	Infant carrying pattern		Description of infant carrying pattern	Litter size*	Sample size
*Cheirogaleus medius*	Carried orally	(16,18,23)	Left in the nest and then carried orally and parked	1–4	3
*Daubentonia madagascariensis*	Carried orally	(16,18)	First two months in the nest, retrieved to the nest orally or herded back	1	4
*Eulemur albifrons*	Fur clingers	(18,23)	Clings to the mother fur	1–2	1
*Eulemur collaris*	Fur clingers	(18,23)	Clings to the mother fur	1–2	3
*Eulemur coronatus*	Fur clingers	(18,23)	Clings to the mother fur	1–2	4
*Eulemur flavifrons*	Fur clingers	(16,18,23)	Clings to the mother fur, carried ventrally first and then dorsally	1–2	7
*Eulemur macaco*	Fur clingers	(16,18,23)	Clings to the mother fur, carried ventrally first and then dorsally	1–2	2
*Eulemur mongoz*	Fur clingers	(16,18,23)	Clings to the mother fur, carried ventrally first and then dorsally	1–2	5
*Eulemur rubriventer*	Fur clingers	(16,18,23)	Clings to the mother fur, carried ventrally first and then dorsally	1–2	6
*Eulemur rufus*	Fur clingers	(16,18)	Clings to the mother fur, carried ventrally for 4 weeks and then dorsally	1–2	2
*Eulemur sanfordi*	Fur clingers	(18)	Clings to the mother fur	1–2	1
*Hapalemur griseus*	Fur clingers	(16,18,23)	First days in the nest with the mother, then carried orally and parked and after one week clings to the mother fur	1	3
*Hapalemur simus*	Fur clingers	(18)	Clings to the mother fur	1	4
*Lemur catta*	Fur clingers	(16,18,23)	Clings to the mother fur, carried ventrally for first few days and then dorsally	1–2	6
*Microcebus murinus*	Carried orally	(16,18,23,34)	Left into the nest at first and then carried orally and parked	1–4	4
*Nycticebus coucang*	Fur clingers	(16,35,36)	Clings to the mother fur for the first 4–6 weeks and then parked	1	1
*Nycticebus pygmaeus*	Fur clingers	(36)	Clings to the mother fur for the first 4–6 weeks and then parked	1–2	3
*Otolemur crassicaudatus*	Carried orally	(16,34,35,37)	Carried by mother orally or dorsally and park for short periods	1–3	1
*Propithecus coquereli*	Fur clingers	(18,23)	Clings to the mother fur, carried ventrally for 6 weeks and then dorsally	1	6
*Varecia rubra*	Carried orally	(18,23,38)	Left into the nest and then carried orally and parked	1–4	6
*Varecia variegata*	Carried orally	(16,18,23,34)	Left into the nest and then carried orally and parked	1–4	6

^*^From DLC pub#1252, Oct 2013.
