# Supplementary material for: Holding-on: co-evolution between infant carrying and grasping behaviour in strepsirrhines
Source: Sci Rep. 2016 Nov 24;6:37729. doi: 10.1038/srep37729 (PMC5121892; doi:10.1038/srep37729)
Supplement: Supplementary Information [file srep37729-s1.doc]

Title: Holding-on: co-evolution between infant carrying and grasping behaviour in strepsirrhines

Louise Peckre, Anne-Claire Fabre, Christine E. Wall, David Brewer, Erin Ehmke, David Haring, Erin Shaw, Kay Welser and Emmanuelle Pouydebat

Supplementary Table S1: Table of the size of the bog and hard static food items per species. The size of the food items is given in centimetres.

|  | *Daubentonia madagascariensis* | *Eulemur albifrons* | *Eulemur collaris* | *Eulemur coronatus* | *Eulemur flavifrons* |
| --- | --- | --- | --- | --- | --- |
| Apple | 8 | 4 | 4 | 4 | 4 |
| Bamboo | - | - | - | - | - |
| Big chow | - | - | 4 | 4 | 4 |
| Broccoli | - | - | 5 | 5 | 5 |
| Carrot | 5-10 | - | 3.5 | 3.5 | 3.5 |
| Cauliflower | - | - | - | - | - |
| Cucumber | 5-10 | 5 | 5 | 5 | 5 |
| Celery | - | 4-8 | - | - | 8 |
| Coconut | 10 | - | - | - | - |
| Green cabbage | - | - | - | - | - |
| Branches | - | - | - | - | - |
| Leek | - | - | - | - | - |
| Medium chow | - | - | - | 3 | - |
| Red cabbage | - | - | - | 4.5 | 4.5 |
| Sweet potato | 5 | - | - | 5 | - |
| Tamarin | 5-10 | - | - | - | - |
| Fennel | - | - | - | - | - |
|  | *Eulemur macaco* | *Eulemur mongoz* | *Eulemur rubriventer* | *Eulemur rufus* | *Eulemur sanfordi* |
| Apple | - | 4 | 4 | 4 | 4 |
| Bamboo | - | - | - | - | - |
| Big chow | 4 | 4 | 4 | 4 | 4 |
| Broccoli | 5 | 5 | 5 | 5 | - |
| Carrot | 3-5 | 3-5 | 3-5 | 3-5 | 3-5 |
| Cauliflower | - | - | - | - | - |
| Cucumber | - | - | 5 | - | - |
| Celery | 8 | 8 | - | - | - |
| Coconut | - | - | - | - | - |
| Green cabbage | - | 4.5 | - | - | - |
| Branches | - | - | - | - | - |
| Leek | - | - | - | - | - |
| Medium chow | - | - | 3 | - | - |
| Red cabbage | 4.5 | 4.5 | 4.5 | 4.5 | - |
| Sweet potato | - | - | - | - | - |
| Tamarin | - | - | - | - | - |
| Fennel | - | - | - | - | - |
|  | *Hapalemur griseus* | *Lemur catta* | *Otolemur crassicaudatus* | *Propithecus coquereli* | *Varecia rubra* |
| Apple | 4 | 4 | 4 | 4 | 4 |
| Bamboo | 10-20 | - | - | - | - |
| Big chow | - | 4 | - | - | 4 |
| Broccoli | - | - | 5 | 5 | - |
| Carrot | 3.5 | 3.5 | - | 6 | 3.5 |
| Cauliflower | - | - | - | 5 | - |
| Cucumber | 5 | 5 | - | 5 | 5 |
| Celery | - | - | - | 8 | 8 |
| Coconut | - | - | - | - | - |
| Green cabbage | - | - | - | - | - |
| Branches | - | - | - | 50 | - |
| Leek | - | - | - | 15 | - |
| Medium chow | - | 3 | - | - | 3 |
| Red cabbage | - | - | - | 4.5 | 4.5 |
| Sweet potato | 5 | - | 5 | 5 | - |
| Tamarin | - | - | - | - | - |
| Fennel | - | - | - | - | - |
|  | *Vareica variegata* | *Cheirogaleus medius* | *Microcebus murinus* | *Hapalemur simus* | *Nycticebus coucang* |
| Apple | 4 | 3 | 2 | 4 | 4 |
| Bamboo | - | - | - | - | - |
| Big chow | 4 | - | - | - | - |
| Broccoli | 5 | - | - | 5 | - |
| Carrot | 3-5 | 2 | 2 | 3.5 | - |
| Cauliflower | - | - | - | - | - |
| Cucumber | 5 | - | - | - | 5 |
| Celery | - | - | - | 7 | - |
| Coconut | - | - | - | - | - |
| Green cabbage | - | - | - | - | - |
| Branches | - | - | - | - | - |
| Leek | - | - | - | - | - |
| Medium chow | 3 | - | - | - | - |
| Red cabbage | 4.5 | - | 3 | - | - |
| Sweet potato | - | - | - | - | - |
| Tamarin | - | - | - | - | - |
| Fennel | - | - | - | 6-10 | - |
|  | *Nycticebus pygmaeus* |  |  |  |  |
| Apple | 4 |  |  |  |  |
| Bamboo | - |  |  |  |  |
| Big chow | - |  |  |  |  |
| Broccoli | - |  |  |  |  |
| Carrot | 3-4 |  |  |  |  |
| Cauliflower | - |  |  |  |  |
| Cucumber | - |  |  |  |  |
| Celery | - |  |  |  |  |
| Coconut | - |  |  |  |  |
| Green cabbage | - |  |  |  |  |
| Branches | - |  |  |  |  |
| Leek | - |  |  |  |  |
| Medium chow | - |  |  |  |  |
| Red cabbage | 4.5 |  |  |  |  |
| Sweet potato | - |  |  |  |  |
| Tamarin | - |  |  |  |  |
| Fennel | - |  |  |  |  |
